# The concurrence of musculoskeletal pain and associated work-related factors: a cross sectional study

**DOI:** 10.1186/s12889-016-3306-4

**Published:** 2016-07-22

**Authors:** Rita de Cássia Pereira Fernandes, Silvana Maria da Silva Pataro, Roberta Brasileiro de Carvalho, Alex Burdorf

**Affiliations:** Departamento de Medicina Preventiva e Social, Faculdade de Medicina da Bahia, Universidade Federal da Bahia, Largo do Terreiro de Jesus, s/n. Centro Histórico, 40.026-010 Salvador, Bahia Brazil; Department of Public Health, Erasmus MC, University Medical Center Rotterdam, P.O. Box 2040, 3000 CA Rotterdam, The Netherlands

**Keywords:** Multi-site pain, Musculoskeletal, Pain, Neck, Upper limbs, Low back, Lower limbs, Co-morbidity, Widespread pain, Workload

## Abstract

**Background:**

Several recent studies have described the presence of musculoskeletal complaints, presenting evidence that multisite musculoskeletal pain (MP) is more often present than single-site musculoskeletal pain. However, less is known about determinants of this multimorbidity, particularly, concerning the role of occupational factors and, mainly, what determines single or multisite pain. This study described the associations between pain in different body sites and investigated related factors to MP in workers from Brazil.

**Methods:**

A total of 1070 workers (228 women and 842 men), from urban cleaning services and from shoe manufacturers, participated in this cross sectional study (response 97 %). Interviewer-administered questionnaire included sociodemographic factors, physical and psychosocial work demands, leisure-time activities and musculoskeletal pain which was presence of pain in previous seven days, considering eight body sites and MP, the sum score of all painful sites, varying 0–8. A factor analysis was performed that captured the nine variables of physical exposure into two latent factors. Associations of pain between different body sites were assessed. Cox regression analyses, presenting the prevalence ratio (PR), showed the related factors to MP.

**Results:**

In the previous seven days, 30 % of workers had MP. For all body sites, comorbidity ranged from 72 % to 91 %. Having pain in one body site is associated with pain in other site and the associations between proximal sites were stronger than between more distal sites. High exposure to manual material handling and awkward postures (PR = 1.5, 95 % CI 1.1–2.0), job strain (PR = 1.2, 95 % CI 1.0–1.6), and low social support (PR = 1.3, 95 % CI 1.0–1.7) and being woman (PR = 1.7, 95 % CI 1.3–2.3) were associated with MP. Risk factors for single–site pain and for subsequent musculoskeletal comorbidity were very similar, suggesting an additive effect of risk factors.

**Conclusions:**

Most workers reported MP that was associated with several work-related factors. The findings support the idea that multisite pain is a continuum of single-site pain, maintained by exposure to several risk factors, rather than the result of a specific risk factor that initiates the multisite pain but not single-site pain. Workplace interventions are needed to decrease the number of pain sites, in order to improve the worker’s health.

## Background

The scientific literature in the past decades has shown the high prevalence of musculoskeletal disorders (MSD) in occupational populations, in industrialized and developing countries. MSD are also an important cause of sickness absence and, in Brazil, ranked the second most prevalent health problem for receiving social benefits for temporary and permanent disability [1].

The main approach applied to study MSD, in these past decades, is based on specific pain in one body site, such as neck pain, pain in upper limbs, and low back pain. Not only the descriptions of the prevalence of pain, as main indicator of MSD, but also the analysis of important determinants of these disorders, have been done according to this approach, i.e., by single site pain.

Nevertheless, in the past few years ago, some authors have discussed that most workers report pain in more than one body site. Several studies have described the presence of multisite pain (MP), both in industrial workers’ population and among service workers [2–16]. There are also several studies showing the importance of this widespread pain in the general population [17–20].

The pioneer studies date from the early 2000s. Natvig et al. [17], found that low back pain along with widespread pain in the previous week was more common than localized back pain among general population in a municipality of Norway. Molano et al. [2], studying a population of scaffolders, discussed the co-morbidity between low back pain and musculoskeletal pain in other body sites. In these pioneer studies, it is hypothesized that “widespread pain could act as a marker of severity for LBP” [17] and “most of the workers not only had back pain but also another musculoskeletal complaint [2]. In 2002, Yeung et al. [3] found that “musculoskeletal symptoms for multiple body parts (two or more) were more prevalent (64 % of all workers) than those for single body regions (19 %). Approximately 85 % of lower back symptoms were associated with disorders in other body regions.” Morken et al. [4] found that widespread pain - in the previous 12 months - predicted short-term and long-term sickness absence among industrial workers. Another study stated that many industrial workers with low back pain experienced musculoskeletal comorbidity in the past 12 months, which was related to a poorer general health and health related quality of life [5]. Alexopoulos et al. [6] reported a high concurrence of musculoskeletal disorders in dentists.

Since then, several studies have been conducted that demonstrated that musculoskeletal multimorbidity has a great impact on overall quality of health, activities of daily living, physical fitness, social activities, reduced work ability and sickness absence than single body segment pain [7–12, 18, 19]. Carnes et al. [19] stated that “of those with chronic musculoskeletal pain, only one in four have single-site pain. Single-site chronic pain is uncommon”. According to Kamaleri et al. [18], “most people having musculoskeletal pain reported pain from a number of sites” and with increased number of pain sites functional problems also increased.

High physical demands, like repetitive movements, awkward postures, heavy lifting, and psychosocial demands have been associated with Multisite Pain. Overweight or obesity and low leisure-time physical activities have been also found as risk factor to MP. Besides, women seems to be more prone to report pain in multiple body sites [8, 9, 16, 20].

In summary, there is clear evidence that multisite pain is much more common than single site pain. However, there is surprising few studies that have addressed underlying risk factors for multisite pain. Therefore, the aims of this study were (i) to estimate the prevalence of multisite pain, (ii) to describe the associations between pain in different body sites and (iii) to investigate related factors to multisite pain in workers from Bahia, Brazil.

## Methods

### Study population

In this cross sectional study, the study population comprised workers from shoe industry companies and workers from urban cleaning services in the state of Bahia, Brazil. These companies were selected for their willingness to provide access for research, the presence of physical demands at work, and being located in the vicinity of the university.

A total of 1070 workers participated in the study (response 97 %). The study population comprised: a stratified random sample from two shoe industry’s companies, proportional to the number of employees in each company and proportional to gender (*n* = 446), in Bahia, in 2012; all urban cleaning workers, the maintenance and operation staff, from the company that provides the service to Salvador City, Bahia, Brazil, in 2010 (*n* = 624). All participants were employed when the study was conducted. In the shoe industry the main occupations involved were assemblers or manufacturers of shoes and machine operators, and occupations in urban cleaning services were garbage collectors, truck drivers, and maintenance workers. In this study population, there is a set of different occupations and duties that varies from physically light duties (8 % of workers), maintenance duties, performed walking or in standing posture, with occasional material handling (8 %), tasks predominantly performed in sitting posture with repetitive movements with hands or arms (23 %) to occupations highly physical-demanding, with frequent handling of heavy materials, while jumping, running or walking behind the truck (34 %), tasks requiring walking while cleaning beaches or urban areas, involving managing tools (8 %) and occupations in assemble lines, assembling and manufacturing shoes, involving a long working day in standing posture with repetitive movements with arms and hands, handling light materials (18 %).

To collect the data, interviews with a structured questionnaire were conducted, by a team of trained interviewers consisting of one health and safety engineer, three physiotherapists, one ergonomist and four academics from the physiotherapy course. They were aware about being sure of clarifying all questions.

Data were collected at each participating company, during a regular working day, in a reserved place, ensuring privacy to workers. Data collection was preceded by meetings with researchers and workers inside each company. The conversation aimed to introduce the research team, inform about the study objectives, about the institution in charge of the research, Federal University of Bahia, a public university, and about the fact that the companies were just contacted to allow the access to the work environment by researchers, but they did not have any participation in the study or access to individual data. This initiative was taken based on respect for the right to information of each research subject and to reduce the bias of information.

### Questionnaire

The questionnaire included sociodemographic factors (age, gender), physical work demands and psychosocial demands, lifestyle habits, leisure-time physical activities and presence of musculoskeletal complaints.

Musculoskeletal complaints were measured by the enlarged version of Nordic Musculoskeletal Questionnaire (NMQ) [21, 22]. Studies of validity and reliability with the NMQ generally revealed high validity and reliability [21, 23, 24]. The questionnaire asked about the presence of pain in the previous 12 months and in the previous week before the interview, for each one of the following body sites: 1. upper limbs (hand, wrist, forearm, elbow), 2. neck, 3. shoulder, 4. upper back, 5. lower back, 6. upper legs/knees, 7. lower legs, 8. Ankles/feet. Musculoskeletal Pain was defined as presence of pain in the previous seven days, considering the body sites mentioned above. Multisite pain was defined by the sum score of all body sites with pain, varying from zero to eight, i. e., based on the eight body sites above, individuals with no pain scored 0, those with pain in one body site scored 1 and so on, up to score 8 for individuals with pain in all eight body sites.

Physical work demands were measured by means of workers’ self-reports on a set of questions using a 6-point scale, ranging from 0 to 5 (scale of duration), with verbal qualifiers at the ends (0 = “never” and 5 = “all the time”). Questions were asked about repetitive hand movements; general working postures like sitting, standing, walking; awkward postures like arms above shoulder height, trunk bent forward or trunk twisted, and squatting; material handling like weight lifting, pulling or pushing; and mechanical grip force on the object of work. For this last variable, we used a 6-point response scale on intensity and verbal qualifiers at the ends were “too weak” = 0 and “too strong” = 5. Factor analysis was carried out to identify underlying factors, reduce the number of variables and prevent variable redundancies.

Psychosocial aspects of work were measured by means of the Job Content Questionnaire (JCQ), with a four point scale, in the version translated into Portuguese and validated by Araújo and Karasek [25], whose Cronbach’s alpha coefficients revealed acceptable internal consistency and factor analysis showed high consistency with the theoretical model. According to this Karasek model [26], workers are supposedly at risk when under high job demands and low job control. Besides, experiencing low social support, either from colleagues or supervisors, will increase health risk. Job demands were measured by nine questions, related to working fast, working hard, excessive work, insufficient time to complete the work, and conflicting demands. Job control was measured by six questions on skills and three questions on authority to make decisions, i.e., nine questions in total. These questions regarded aspects such as required skills, task variety, learning new things, and amount of repetitive work. Sum scores across job control and job demands were dichotomized at median scores to define high strain (high demand and low control). Besides, social support was analyzed by combining both variables, support from colleagues and from supervisors, in one variable, dichotomized by median scores.

With regard to leisure-time physical activities, interviewers asked individuals about what they usually do while not working in the company or at home. This question had a four-item response scale: 1. Competitive sports activity, 2. Running, doing gymnastics, swimming, playing football, bike riding, 3. Walking, fishing and gardening, 4. Reading the newspaper or a magazine, watching television and studying. Individuals with answers 1, 2 or 3 were considered as “active” in leisure-time.

Body Mass Index (BMI, Kg/m^2^) was calculated based on direct measurements of height and weight done by interviewers. It was considered as low weight <18.5, normal 18.5–25, overweight > =25–30 and obesity > = 30 kg/m^2^. A dichotomized variable was used: normal <25 or overweight/obesity > =25.

### Statistical analysis

The statistical analysis consisted of several steps. First, a factor analysis was performed that captured the nine variables of physical exposure into two latent factors that explained 63 % of the total variance. The initial extraction was made through the main components of the model, and factors were obtained without rotation. Their composition, in a decreasing order of the loads presented by each variable, was as follows: Factor 1 (Initial Eigenvalue = 4.494; variance = 49.9 %) characterized physical demands of material handling and strenuous postures including awkward postures: pulling, lifting and pushing weights, squatting, arms above shoulder height, trunk bending, trunk rotation, mechanical hand pressure on the object of work. Factor 2 (Initial Eigenvalue = 1.186; variance = 13.2 %) characterized repetitive work with the variable “repetitive hand movements”. Both factors were used as independent variables for physical work demands with cut-off point at third quartile values.

In the second step, musculoskeletal multisite pain was described, estimating its prevalence and describing the proportions of concurrent pain for each one of the eight body sites. The associations between pain in different body sites were assessed, presenting the prevalence ratio (PR) as measure of association and 95 % confidence interval.

In the third step, Cox regression analyses were performed to investigate the factors associated with multisite pain. The PR is a better approximation of the risk than the often-used odds ratio in a situation with a high prevalence of disease. The Cox regressions consisted of three different models. In the first model, multisite pain is compared with no pain. In the second model single-site pain is compared with no pain and in the third model, multisite pain is compared with single-site pain. All models were adjusted for age, gender, body mass index, physical demands at work, psychosocial work demands and leisure-time physical activities. The differences between these three models allow interpretation on the absence or presence of synergistic effects of risk factors, i.e., without synergy the influence of a particular risk on multisite pain (model 1) would equal the combined effect of that risk factor on single-site pain (model 2) and on subsequent multisite pain (model 3).

The logistic regression analysis is widely used in cross sectional studies and provides odds ratios estimates. Odds Ratios typically overestimate the associations for outcomes that are frequent, such as MSD. So, we conducted Cox regression analysis, based on Coutinho et al. [27], in order to provide Prevalence Ratio estimates. In analyses of data from cross-sectional studies, the Cox and Poisson models with robust variance are better alternatives than logistic regression analysis. Besides, these authors have shown that if in cross sectional studies a constant risk period is assigned to all the individuals in the study, the hazard ratio in Cox regression analysis equals the PR.

The use of statistical estimation in this study is only aimed at showing the precision of estimation, by means of the confidence intervals of 95 %. Our study population is not a random sample, so, the inferential statistics presented serves merely “as a minimum estimates of the actual uncertainty about the object of estimation” [28]. We assume a critical view about statistical inference, given the common misinterpretation of statistical information [28, 29].

All descriptive and inferential statistical analyses were performed using the SPSS software, Version 21.

## Results

The study population of 1070 workers comprises 228 women and 842 men, with a mean age of 31 years (25th percentile = 26 years, 75th percentile = 37 years).

Table 1 shows that the prevalence of musculoskeletal pain varied from 10.4 % for ankle/feet to 23.8 % for lower back. The prevalence of single-site pain without any musculoskeletal co-morbidity was much lower, varying between 1.1 % for neck pain to 6.6 % for lower back pain. For all body sites, musculoskeletal co-morbidity was high. Among those with neck pain, 43.7 % had concurrent pain in upper back and 34 % had shoulder pain. In total, the proportion of neck pain concurrent with pain in at least one other site is 90.5 %, i.e., almost all workers with neck pain reported pain in another body site. The same pattern was observed for other musculoskeletal complaints.

**Table 1:** Co-occurrence of pain based on conditional proportions of pain in previous seven days, among industrial workers and urban cleaning service workers, Bahia, Brazil

Body site	Prevalence		No pain in other sites		Concurrent pain during the previous seven days (%). Conditional proportions.							
	n	%	n	%	Neck	Shoulder	Upper back	Upper limbs	Low back	Upper leg/knee	Lower Leg	Ankle/Feet
Neck	126	11.8	12	1.1	-	34.1	43.7	41.3	53.2	28.6	40.5	27.8
Shoulder	131	12.2	22	2.1	32.8	-	38.2	39.7	51.9	29.8	35.1	29.0
Upper back	151	14.1	25	2.3	36.4	33.1	-	37.7	50.3	31.8	40.4	27.2
Upper limbs	198	18.5	47	4.4	26.3	26.3	28.8	-	39.9	27.8	36.4	24.2
Low back	255	23.8	71	6.6	26.3	26.7	29.8	31.0	-	27.1	28.6	20.8
Upper leg/ knee	151	14.1	32	3.0	23.8	25.8	23.8	36.4	45.7	-	37.7	33.1
Lower Leg	173	16.2	28	2.6	29.5	26.6	35.3	41.6	42.2	32.9	-	37.0
Ankle/Feet	111	10.4	16	1.5	31.5	34.2	36.9	43.2	47.7	45.0	57.7	-

In the previous seven days, 23.6 % of workers had single-site pain and 30.1 % of workers had pain in more than one site. Among those who had pain, 56.0 % had pain at least in two sites (Table 2).

**Table 2:** Prevalence of pain by number of sites in previous seven days, among industrial workers and urban cleaning service workers, Bahia, Brazil

Number of Body Sites	Pain in the previous seven days		
	n	%	Cumulative percent
None	495	46.3	46.3
Single	253	23.6	69.9
Two	135	12.6	82.5
Three	87	8.1	90.7
Four	47	4.4	95.0
Five	19	1.8	96.8
Six	16	1.5	98.3
Seven	11	1.0	99.3
Eight	7	0.7	100.0
Total	1070	100.0	

For all eight body sites, musculoskeletal comorbidity was high, varying between 72.2 % for lower back to 90.5 % for neck pain. Many workers with musculoskeletal comorbidity reported pain in more than three sites (Table 3).

**Table 3:** Distribution of single-site pain and multisite pain among those with any musculoskeletal complaint in the previous seven days, among industrial workers and urban cleaning service workers, Bahia, Brazil

Body site	Number of pain sites		
	1	2–3	>4
	%	%	%
Neck	9.5	46.9	43.6
Shoulder	16.8	38.2	45.0
Upper back	16.6	39.8	43.6
Upper limbs	23.7	42.4	33.9
Low back	27.8	42.0	30.2
Upper leg/ knee	21.2	39.7	39.1
Lower Leg	16.2	42.2	41.6
Ankle/Feet	14.4	34.2	51.4

Table 4 shows that having pain in one body site is associated with pain in other site. For example, the prevalence of neck pain among who had shoulder pain was 3.7 times that prevalence among those without the shoulder pain. The associations between proximal sites were stronger than between more distal sites with pooled PRs of 3.75 and 2.87, respectively. For example, the association between lower leg pain and ankle/feet pain (PR = 7.1) was higher than lower back pain with ankle/feet pain (PR = 2.9). Likewise, upper back pain had a stronger association with neck pain (4.7) than with upper limbs pain (PR = 2.5).

**Table 4:** Prevalence ratios (PR) of pain in a site relative to another in previous seven days, among industrial workers and urban cleaning service workers, Bahia, Brazil

Site	Neck^a^	Shoulder^a^	Upper back^a^	Upper limbs^a^	Low back^a^	Upper leg/knee	Lower leg^a^	Ankle/Feet^a^
Neck^b^	-	3.7(2.7–5.0)	4.3(3.3–5.6)	2.7(2.1–3.4)	2.7(2.2–3.3)	2.3(1.7–3.2)	3.1(2.4–4.1)	3.4(2.4–4.9)
Shoulder^b^	3.7(2.7–5.1)	-	3.5(2.7–4.7)	2.5(2.0–3.3)	2.6(2.1–3.2)	2.5(1.8–3.4)	2.6(1.9–3.4)	3.7(2.6–5.3)
Upper back^b^	4.7(3.5–6.4)	3.8(2.8–5.1)	-	2.5(1.9–3.2)	2.6(2.1–3.2)	2.8(2.1–3.8)	3.3(2.5–4.3)	3.6(2.5–5.0)
Upper limbs^b^	3.1(2.2–4.3)	2.9(2.1–4.0)	2.7(2.0–3.6)	-	2.0(1.6–2.4)	2.5(1.9–3.4)	3.1(2.4–4.1)	3.3(2.4–4.7)
Low back^b^	3.6(2.6–5.0)	3.4(2.5–4.7)	3.2(2.4–4.3)	2.1(1.7–2.7)	-	2.7(2.0–3.6)	2.3(1.8–3.0)	2.9(2.1–4.1)
Upper leg/knee^b^	2.4 (1.7–3.4)	2.6(1.8–3.6)	2.8(2.1–3.8)	2.3(1.8–3.0)	2.3(1.8–2.8)	-	3.0(2.3–3.9)	5.0(3.6–6.9)
Lower Leg^b^	3.5(2.6–4.8)	2.8(2.0–3.9)	3.5(2.6–4.7)	3.0(2.3–3.8)	2.1(1.7–2.6)	3.1(2.4–4.2)	-	7.1(5.0–9.9)
Ankle/Feet^b^	3.3(2.4–4.6)	3.5(2.6–4.9)	3.2(2.4–4.3)	2.8(2.1–3.6)	2.3(1.8–2.8)	4.3(3.2–5.6)	5.1(4.0–6.4)	-

PR (95 % CI). ^a^Treated as the dependent variable. ^b^Treated as the independent variable

In the first model, being highly exposed to physical work demands, i.e. to manual material handling and awkward postures, increased 1.5 fold the prevalence of multisite pain (95 % CI 1.1–2.0). Job strain (PR = 1.2, 95 % CI 1.0–1.6) and low social support (PR = 1.3, 95 % CI 1.0–1.7) were other occupational factors positively associated with having pain in at least two sites of the body. Women (PR = 1.7, 95 % CI 1.3–2.3) more often had multisite pain than men. The comparison with the second and third model showed that the risk factors for multisite pain are primarily an additive effect of the risk for having a single-site pain (model 2) and the risk for a subsequent comorbidity (model 3). The observed associations for risk factors with single–site pain and with subsequent comorbidity were very similar, also suggesting an additive effect of risk factors (Table 5).

**Table 5:** Multivariate analysis of related factors to multisite and single-site pain in previous seven days among industrial workers and urban cleaning service workers, Bahia, Brazil

Associated factors	Multisite pain vs No pain (*n* = 759)	Single-pain vs No pain (*n* = 693)	Multisite pain vs Single-site pain (*n* = 538)
	PR 95 % CI	PR 95 % CI	PR 95 % CI
High exposure to material handling and awkward postures	1.5 (1.1–2.0)	1.2 (0.8–1.6)	1.3 (0.9–1.7)
High repetitive movements	1.2 (0.9–1.6)	1.2 (0.8–1.6)	1.1 (0.8–1.4)
High strain: High psychological demand and low job control	1.2 (1.0–1.6)	1.2 (0.9–1.7)	1.0 (0.8–1.3)
Low social support	1.3 (1.0–1.7)	1.3 (1.0–1.7)	1.0 (0.8–1.3)
Gender: female	1.7 (1.3–2.3)	1.4 (1.0–2.0)	1.3 (0.9–1.7)
Overweight	1.1 (0.9–1.4)	1.2 (0.9–1.6)	0.9 (0.8–1.2)
Above 37 yrs	0.9 (0.7–1.2)	0.9 (0.7–1.2)	1.0 (0.8–1.3)
No leisure-time physical activity	1.0 (0.8–1.3)	1.0 (0.8–1.4)	1.0 (0.8–1.3)

## Discussion

The aims of this study were to estimate the prevalence of multisite pain, to describe the associations between pain in different body sites and to investigate related factors to multisite pain in workers from Bahia, Brazil. We found that in the previous seven days, one third of workers had multisite pain and having pain in one site was highly associated with pain in another body site. Workers under high exposure to physical demands at work, such as manual material handling and strenuous postures, had the highest prevalence of pain, regardless being men or women.

The study was conducted in workplaces during working days, in which the workers were interviewed about multisite body pain. In those days, they were performing their tasks, either in cleaning urban services or in the shoe factories. This study corroborates that concurrent musculoskeletal pain is higher than single-site pain for all body sites in the short time period of seven days. For every pain site there was at least one other site of pain in a proportion of 72 % up 90 %. This finding is compatible to Kamaleri et al. [18], who stated that “for every pain site more than 85 % had pain from at least one other region”. The results showed a much higher proportion of concurrent pain than single site-pain, hence, single-site pain seems to be a rare outcome among workers. This was also observed in the general population, where multisite pain seems to be much more frequent than a single-site pain [19]. In this latter study among adults registered with general practitioners in England, the prevalence of single-site pain in the past 12 months was very low, like in the present study, ranging from 1 to 3 % versus 1 to 7 % in our study.

Comparisons between our findings and those by Kamaleri et al. [18], who assessed pain for the same period of time, i.e., seven days, reveal that proportion of single site pain is equally much lower than that of multisite pain.

The high proportion of concurrent pain, based on eight separated body sites, might be the consequence of the high prevalence of pain in different sites. Croft et al. [30] state that associations between pain complaints will inevitably occur because of the underlying frequency of the individual syndromes. To those authors, no one “should expect that any specific concurrence would happen more often than expected (…) given that any pain is more likely to occur in the presence of another pain, whatever the location of pain: anyone setting out to study a particular association between two pain syndromes can be both reassured (you will find it) and cautioned (there will be nothing special about the result)”. Our results are compatible with this statement, in the sense that the prevalence of concurrent pain was high for every pain site. The prevalence ratios were high regarding the association among pain in different sites, i. e., having pain in one site was associated with an increased occurrence of pain at another site. We did find various associations between two pain-sites, and, in general, proximal sites were higher associated than distal ones.

Haukka et al. [12], who analyzed associations between pain in different body sites among female workers, found that the prevalence of neck pain among those who had back pain to be 1.4 times the prevalence of neck pain among those without back pain and the association was 1.6 times among back pain and upper limbs’ pain. Alexopoulos et al. [6], studying female and male dentists, found that having low back pain resulted in 3 fold chance of having neck pain and 2.4 fold chance of having pain in hand/wrist. These latter results are very similar to ours, 3.6 and 2.1, respectively.

Several studies have discussed the presence of concurrent musculoskeletal pain and its repercussion on health related quality of life, on sick leave [5, 11, 13, 18] and on loss of productivity or work disability [7, 10, 14, 15]. However, less is known about determinants of this multimorbidity, particularly, concerning the role of occupational factors and, mainly, what determines single or multisite pain.

According to our findings, having concurrent pain, i.e., presenting a multisite pain, was associated with being exposed to high physical demands at work. Also, we found a contribution of low job support to the occurrence of multisite pain and working under high psychological demand without a good job control increased the prevalence of having multisite pain.

The high prevalence of multisite pain in our population is compatible with findings of studies among different occupational groups, like kitchen workers [12] or food industry workers [15] or dentists [6], and compatible with results in the general population [18, 19]. This main finding of our study indicates that single site pain is a rare condition compared to concurrent pain. This supports the idea that when considering different patterns of exposure to physical demands at work, one should bear in mind that the occurrence of musculoskeletal pain most often will not be localized in one specific site. Hence, exposure-response relationships may be less specific than observed. It is important to consider the questions raised by Croft at al. [30] about the nature of central pain and pain processing, and about the plastic memory of the nervous system for pain that operates independently of the initial site of nociceptive stimulus. This theory of central pain is helpful to explain why the associations between pain in different body locations, whatever is the location, were always strong according to our results.

The workers in our study are exposed to a diversity of physical demands at work. Our study population comprises many occupations: office workers in shoe manufacturing companies, assemblers or manufacturers of shoes and machine operators, garbage collectors, truck drivers, and maintenance workers. The tasks performed by workers involve pulling, lifting and pushing weights, squatting, arms above shoulder height, trunk bending, trunk rotation, mechanical hand pressure on the object of work, and, for most of workers, a high exposure to repetitive hand movements. The diversity of physical demands in our population and different patterns of exposure may be an important reason for presence of multisite pain.

In this study, with a high prevalence of multisite pain in the last seven days, the pattern of physical demands exposure among workers varied from more localized physical demand on specific body sites to uniformly distributed physical demands on the total body as described above. Regardless the set of different pattern of physical exposure, our results are compatible with studies in specific occupations, such as Haukka et al. [12] who reported in a study among female kitchen workers also a high prevalence of multisite pain. Alexopoulos et al. [6] studied dentists, also a specific occupation, and found a high prevalence of concurrent pain. It is possible to say that the pattern of physical demands at work for a dentist is very different of the exposure pattern of a worker in dynamic work, with handling heavy materials in cleaning services. Therefore, there is no single or obvious explanation to the high prevalence of multisite pain as a direct consequence of physical work load on the painful body sites. Hence, we share the discussion by Croft et al. [30] based on two important explanations of the high occurrence of multisite pain: first, a general vulnerability related to the central pain processing, and, second, shared risk factors that concern the role of physical demands at work that simultaneously act at more than one body site

It is hypothesized that based on the shared biomechanical risks, or physical exposure, occupational situations are more frequently demanding for a body region involving closer sites, at least, closer sites will be firstly reached by damages. Even though, dependent on the physical demands pattern, we can expect not only association among pain in closer sites but also association among farther ones. For example, pain in lower back and shoulder pain might be associated when both body sites experience high mechanical load. Another example is the large amount of tasks performed on countertops or tables (on assembly lines) that involves standing posture and neck bending or holding head bent forward – this fact can result in high physical demands to lower limbs, but, at the same time, it results in neck and upper back demanding. So, not only the closest body sites but also the farthest sites will be affected as consequences of the exposure to physical demands.

In an earlier prospective study, Macbeth et al. [31] investigated related factors to widespread pain in adults, selected from a population-based primary care register. They presented evidence of an association between repetitive movements with wrists, pushing/pulling heavy weights and kneeling with widespread pain. Harkness et al. [32] found associations between pulling load, squatting, monotonous work, and low support from colleagues with the presence of widespread chronic pain.

Neupane et al. [16], reported associations between multisite pain and repetitive work and awkward posture. Besides, they found a role of the job dissatisfaction and of the poor opportunities to exert influence on executing work activities. Strenuous physical activities were associated with multisite pain in study by Solidaki et al. [8]. Haukka et al. [9] found that perceived physical workload and adverse psychosocial factors at work were also associated with multisite pain.

Those former findings support the results of the current study. The contribution of our study is not only in demonstrating the presence of the high magnitude of multisite pain, even in a short time window like the previous week which present reliable information about concurrent pain, but also in illustrating the important role of work conditions for multisite pain. Beyond, the findings support the idea that multisite pain is a continuum of single-site pain, maintained by exposure to several risk factors, rather than the result of a specific risk factor that initiates the multisite pain but not single-site pain. So, our results are compatible with the statement by Croft [33] about considering “number of pain sites as a continuously distributed marker of risk for poor health outcomes”.

There is some evidence about the association between gender and multisite pain. The female population not only has a high prevalence of musculoskeletal pain, but they also seem to report more sites with pain than the male population. The role of gender in determining widespread pain in persons with low back pain has been highlighted since 2001, by Natvig et al. [17], in a study with a general population, and by Carnes et al. [19], who found 20 % less multisite pain among men than among female people. That is compatible with our finding that women more often had multisite pain than men.

The literature refers about the role of somatization to predict chronic widespread pain but this finding is not consistent among studies. Some authors state that most of subjects with multisite pain are not ‘somatizers’ [10]. In studies that have shown associations of somatization with multisite pain, the importance of physical work demands as independent risk factor for musculoskeletal multisite pain has been confirmed [8, 31].

Prevalence of musculoskeletal pain in different body sites will be higher when the recall period of pain is higher. In a longer period of time, the reports of pain from more than one body site (multisite pain) can represent, in the truth, different episodes of pain occurring in different time, instead of being concurrent pain. Since this study aimed to describe multisite pain, the previous seven days may represent a more appropriate period of time to measure simultaneous pain occurrence.

Some strengths of this study, in spite of its cross sectional design, are related to strategies adopted in order to minimize the information bias. Taking into account the context of labor relations in Brazil, the methods applied in our study ensured absolute privacy and confidentiality and information was collected by independent researchers. This will have facilitated the high participation and the trust of workers in the public and respectful institution responsible for the study (Federal University of Bahia). Besides, doing the data collection by means of interviewer-administered questionnaire with a high-qualified team, conscious about interview techniques, work context, labor relations, cultural and linguistic issues of labor environment, contributed to the high response. Moreover, to collect data by means of interview can be a strong point of our study, in order to avoid information bias, because we could clarify any item of the questionnaire when necessary, what certainly improved the quality of workers’ responses. Interviewers had to make sure that workers had understood all information about confidentiality, trying to clarify all possible questions regarding the questionnaire and obtaining the best responses. In spite of the sample size of this study, all these procedures were thoroughly performed.

## Conclusions

Multisite pain has a high magnitude among workers. It affects the daily life of workers and is associated with manual material handling and strenuous body postures in a demanding psychosocial workplace. The findings support the idea that multisite pain is a continuum of single-site pain, maintained by exposure to several risk factors, rather than the result of a specific risk factor that initiates the multisite pain, but not single-site pain. Workplace interventions, with an appropriate approach also regarding the role of gender on this morbidity, are needed in order to decrease the number of pain sites and to improve the quality of life at work. For future studies, it is advisable to not solely focus on musculoskeletal pain in one specific body site. It seems promising measuring multisite pain and to analyze the associated factors, and its consequences related to sickness absence and work ability.

## Abbreviations

BMI, body mass index; JCQ, job content questionnaire; MP, multisite musculoskeletal pain; MSD, musculoskeletal disorders; NMQ, nordic musculoskeletal questionnaire
