# Erratum to: The concurrence of musculoskeletal pain and associated work-related factors: a cross sectional study

**DOI:** 10.1186/s12889-016-3795-1

**Published:** 2016-10-31

**Authors:** Rita de Cássia Pereira Fernandes, Silvana Maria Santos Pataro, Roberta Brasileiro de Carvalho, Alex Burdorf

**Affiliations:** 1 Departamento de Medicina Preventiva e Social, Faculdade de Medicina da Bahia, Universidade Federal da Bahia, Largo do Terreiro de Jesus, s/n. Centro Histórico, 40.026-010 Salvador, Bahia Brazil; 2 Department of Public Health, Erasmus MC, University Medical Center Rotterdam, P.O. Box 20403000 CA Rotterdam, The Netherlands

## Erratum

After publication of the original article [1] it was brought to our attention that author Silvana Maria Santos Pataro was incorrectly included as Silvana Maria da Silva Pataro. The correct spelling of the name is included in the author list of this erratum.
